# Use of flash glucose monitoring for post-bariatric hypoglycaemia diagnosis and management

**DOI:** 10.1038/s41598-020-68029-8

**Published:** 2020-07-06

**Authors:** Carolina B. Lobato, Sofia S. Pereira, Marta Guimarães, Tiago Morais, Pedro Oliveira, Jorge P. M. de Carvalho, Mário Nora, Mariana P. Monteiro

**Affiliations:** 1 Endocrine, Cardiovascular & Metabolic Research, Unit for Multidisciplinary Research in Biomedicine (UMIB), University of Porto, Jorge Viterbo Ferreira 228, Ed.1, 3rd Floor, 4050-313 Porto, Portugal; 2 Department of Anatomy, Institute of Biomedical Sciences Abel Salazar (ICBAS), University of Porto, Jorge Viterbo Ferreira 228, Ed.1, 3rd Floor, 4050-313 Porto, Portugal; 3 Department of General Surgery, Centro Hospitalar de Entre o Douro e Vouga, 4520-211 Santa Maria da Feira, Portugal; 4 Department of Population Studies, ICBAS, University of Porto, 4050-313 Porto, Portugal; 5 Department of Mathematics, Faculty of Sciences, University of Porto, 4169-007 Porto, Portugal

**Keywords:** Obesity, Endocrinology

## Abstract

Our aim was to assess the potential of flash glucose monitoring (FGM) for diagnostic workup of suspected post-bariatric hypoglycaemia (PBH). Patients (N = 13) with suspected PBH underwent a food and symptoms diary (FSD) record along with FGM over 14 days. Targeted data analysis confirmed the occurrence of low glucose events in parallel to meal-triggered symptoms. Glycaemic variability, as assessed by Mean Absolute Glucose change (MAG change), was increased, while a higher risk of glycaemic excursions towards both hyper and hypoglycaemia (ADRR_FGM_GT) was observed in those with more frequent and severe hypoglycaemia. The herein described hypoglycaemia risk index (LBGI_FGM_GT) with a cut-off value of 4.6 showed to have 100% sensitivity and 100% specificity for PBH. This pilot proof-of-concept study highlighted that FSD coupled with FGM followed by targeted data analysis, provides relevant insights towards PBH diagnosis and grading in a user-friendly and easy to implement study protocol. Furthermore, LBGI_FGM_GT demonstrated to be an excellent index for PBH diagnosis. The unexpected improvement of glucose profile noticed along the monitoring time also unravels a possible application for PBH management.

## Introduction

Bariatric surgery is the most effective treatment currently available for patients with morbid obesity, which was proved to achieve long-term weight loss and sustained remission of obesity-related comorbidities^1,2^. Despite benefits of bariatric surgery largely surpass the risks of the procedures, early and late complications can still occur^3^.

Post-bariatric hypoglycaemia (PBH) is a rare yet emerging clinical condition that was first consistently described in 2010^4^. PBH has been mainly reported as a late complication of Roux-en-Y gastric bypass (RYGB)^5–8^ although occasionally presenting after other bariatric surgery procedures^9–12^. PBH is characterized by the occurrence of recurrent postprandial hypoglycaemic events in the presence of normal fasting glucose^8,13,14^ and has the potential to impair considerably the patients’ well-being and quality of life^15^.

PBH diagnosis is currently grounded on the exclusion of other causes for hypoglycaemia in a patient previously submitted to bariatric surgery, since there are no established diagnostic criteria currently available^8^. In addition, despite many hypotheses have been raised, the aetiology of PBH remains uncertain^5,7,8^.

Identifying PBH as a possible cause for post-bariatric patient complaints can be challenging, as hypoglycaemia can present as a large spectrum of unspecific clinical features, including autonomic and neuroglycopenic symptoms such as tremor, sweating, loss of consciousness and even seizures^8,14^, which depending of the predominant signs can lead the clinicians to consider several different conditions in the differential diagnosis ranging from dumping syndrome to epilepsy^4^.

Depending on the criteria used for diagnosis, PBH prevalence can range from 0.2% if based on hospitalization records due to hypoglycaemia^4^ to 6.6% if grounded on symptomatic reports only^16^, so the actual prevalence of PBH is difficult to ascertain. Therefore, an elusive clinical presentation along with the absence of established criteria for PBH diagnosis or clinical management guidelines make this condition particularly challenging.

Our aim was to assess the potential for the use of flash glucose monitoring (FGM) with targeted data analysis in the clinical workup of patients with PBH, based on the outcomes of its implementation in a pilot proof-of-concept study.

## Results

Subjects (N = 13) previously submitted to RYGB surgery that self-reported symptoms suggestive of hypoglycaemia were divided into two sub-groups *No PBH* (interstitial fluid glucose [IFG] < 54 mg/dl < 1%) and *PBH* (IFG < 54 mg/dl ≥ 1%) according to FGM profile. Detailed patient case descriptions are presented to illustrate the two different conditions despite similar clinical presentations.

### Anthropometric, metabolic and demographic patient characteristics

Before surgery, all subjects fulfilled the international clinical criteria to undergo bariatric surgery for primary treatment of obesity and related comorbidities^2^. No significant differences in anthropometric and metabolic parameters were observed between patient subgroups before or after surgery (*p* > 0.05) (Table 1).

**Table 1:** Demographic features, patients comorbidities and anthropometric and biochemical profiles of the patient study group, according to FGM profile *No PBH* (IFG < 54 mg/dl < 1%) and *PBH* (IFG < 54 mg/dl ≥ 1%), based on established criteria for level 2 hypoglycaemia.

	No PBH	PBH	*p* value
**N (% of total)**	5 (38.5%)	8 (61.5%)	–
**Demographic features at evaluation**			
Age (years)	47.6 (42.1–51.9)	50.7 (44.9–55.2)	0.724
Sex (F/M)	5 F/0 M	6 F/2 M	0.487
**Comorbidities before the surgery**			
Obesity (yes/no)	5/0 (100%)	8/0 (100%)	> 0.999
Type 2 diabetes (yes/no)	2/3 (40%)	0/8 (0%)	0.128
Hypertension (yes/no)	3/2 (60%)	4/4 (50%)	> 0.999
Dyslipidaemia (yes/no)	2/3 (40%)	2/6 (25%)	> 0.999
**Anthropometric features at evaluation**			
Time since RYGB (years)	4.8 (2.4–8.2)	6.0 (4.0–6.7)	0.724
Pre-operative weight (kg)	86.0 (81.1–116.5)	105.0 (92.8–115.5)	0.127
Post-operative weight (kg)	65.9 (61.0–80.0)	71.0 (65.0–78.5)	0.594
Pre-operative BMI (kg/m^2^)	36.9 (35.2–44.9)	39.1 (35.8–45.7)	0.833
Post-operative BMI (kg/m^2^)	28.9 (26.3–31.0)	26.4 (24.2–29.4)	0.354
EBMIL (%)	71.5 (67.1–87.5)	89.7 (79.4–109.7)	0.171
**Biochemical profile at evaluation**			
HbA_1c_ (%/mmol/mol)	5.5 (5.0–5.9)/36.6 (30.6–41.0)	5.4 (5.0–5.5)/35.0 (31.7–36.6)	0.598
Glucose (mg/dl/mmol/l)	90.0 (84.0–96.0)/5.0 (4.7–5.3)	97.0 (87.8–104.3)/5.4 (4.8–5.8)	0.271
Insulin (pmol/l)	36.7 (34.6–50.6)	27.4 (24.7–40.0)	0.088
HOMA2-%*β* (%)	76.5 (67.2–97.2)	50.8 (45.7–90.7)	0.127
HOMA2-%S (%)	145.2 (107.7–152.9)	186.1 (137.8–216.0)	0.093
HOMA2-IR	0.69 (0.66–0.94)	0.54 (0.46–0.73)	0.093

Biochemical measurements were performed in plasma samples obtained after an overnight fast. Results are presented as proportions (percentage) and median (interquartile range).

*PBH* post-bariatric hypoglycaemia, *RYGB* Roux-en-Y gastric bypass, *EBMIL* excess BMI loss, *HbA*_*1c*_ glycated haemoglobin, *HOMA2-%β* updated homeostasis model assessment for β-cell function, *HOMA2-%S* updated homeostasis model assessment for insulin sensitivity, *HOMA2-IR* updated homeostasis model assessment for insulin resistance.

### Food and symptoms diary and flash glucose monitoring targeted data analysis

FGM and food and symptoms diary (FSD) data were analysed for a median total time corresponding to sensor lifetime (14 days) subtracted of the first 48 h and the time of data loss due to missed scanning within an 8-h interval (*No PBH*: 11.87 [11.78–11.91] days, median [P25–P75]; *PBH*: 11.90 [11.80–11.95]) (Table 2).

**Table 2:** Study sub-groups’ flash glucose monitoring (FGM) data analysis, according to FGM profile *No PBH* (IFG < 54 mg/dl < 1%) and *PBH* (IFG < 54 mg/dl ≥ 1%), based on established criteria for level 2 hypoglycaemia.

FGM evaluation analysis	No PBH	PBH	*p* value
**N (% of total)**	5 (38.5%)	8 (61.5%)	–
Duration (days)	11.87 (11.78–11.91)	11.90 (11.80–11.95)	0.524
Valid readings (N)	1,115 (1,103–1,123)	1,104 (1,039–1,131)	0.803
Data capture rate (%)	98.83 (97.71–99.51)	97.39 (91.36–99.42)	0.284
MAG change (mmol/l × h^−1^)	2.8 (2.3–2.9)	2.2 (1.6–2.9)	0.354
LBGI_FGM_GT	3.0 (2.6–3.6)	13.0 (11.6–18.1)	**0.002**
HBGI_FGM_GT	2.6 (1.8–3.2)	0.8 (0.4–2.1)	**0.045**
ADRR_FGM_GT	50.2 (39.1–56.1)	85.8 (70.2–91.2)	**0.003**
CONGA1	2.5 (2.0–2.6)	1.9 (1.4–2.6)	0.354
MODD	1.5 (1.1–1.8)	1.2 (0.9–1.5)	0.171

Results are presented as median (interquartile range).

*PBH* post-bariatric hypoglycaemia, *MAG* *change* mean absolute glucose change, *LBGI*_*FGM*_*GT* low blood glucose index (adjusted), *HBGI*_*FGM*_*GT* high blood glucose index (adjusted), *ADRR*_*FGM*_*GT* average daily risk ratio (adjusted), *CONGA1* continuous overlapping net glycaemic action, *MODD* mean of daily differences.

*p* values representative of statistically significant differences (*p* < 0.05) were highlighted in bold.

FGM data analysis revealed that short-term (mean absolute glucose change [MAG change]^17^), intra-daily (continuous overlapping net glycaemic action [CONGA1]) and inter-daily glucose variability (mean of daily differences [MODD]) patterns were similar in both sub-groups (Table 2).

**Figure 1:**
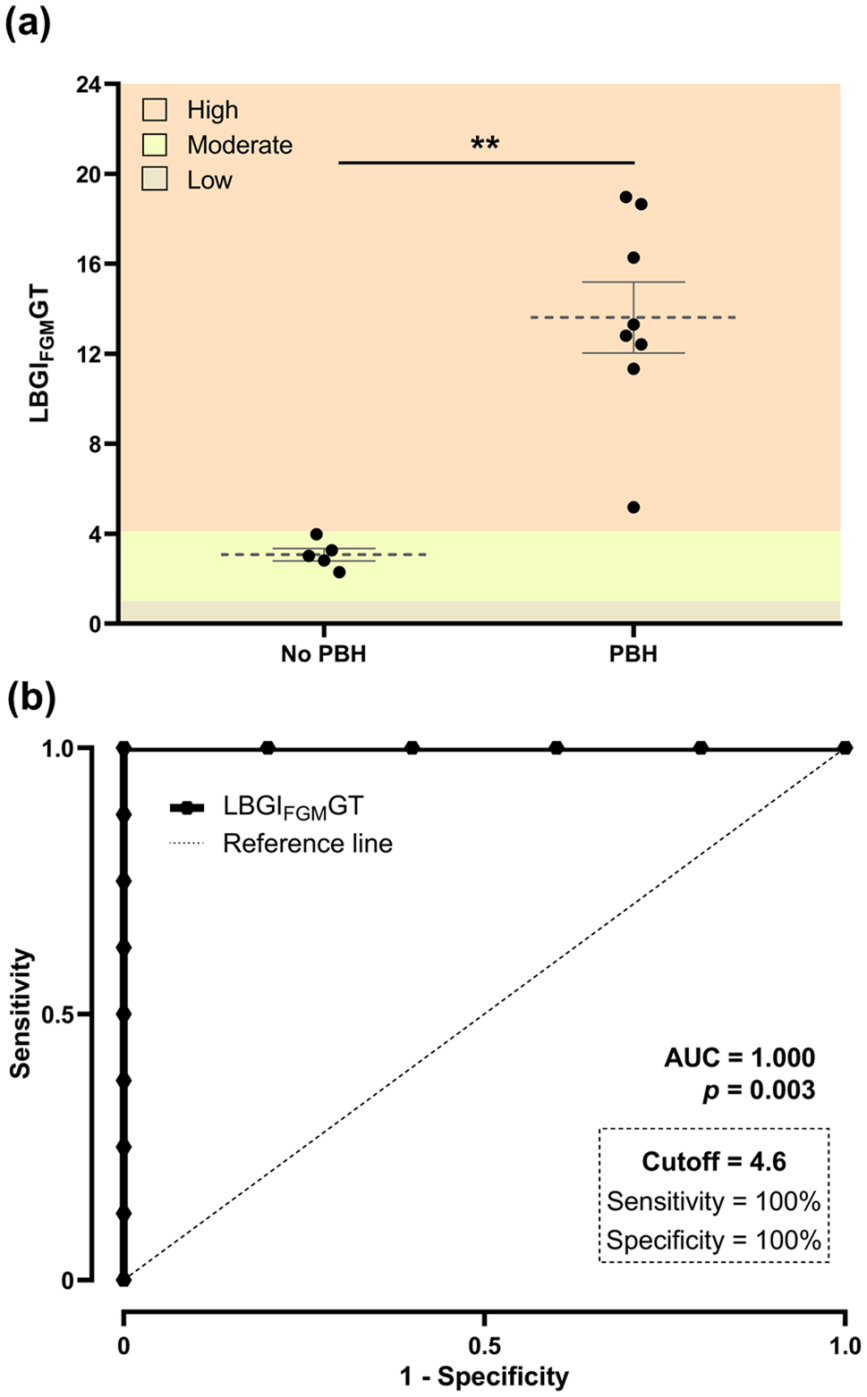
(**a**) Individual LBGI_FGM_GT of the patient study group (N = 13), according to FGM profile *No PBH* (IFG < 54 mg/dl < 1%, n = 5) and *PBH* (IFG < 54 mg/dl ≥ 1%, n = 8), based on established criteria for level 2 hypoglycaemia; Data is represented as mean ± standard error of the mean;**p < 0.01. (**b**) ROC curve analysis of the value of LBGI_FGM_GT (ROC curve AUC = 1.000; *p* = 0.003) to assess BPH severity. At the optimal cut-off value of 4.6, LBGI_FGM_GT had 100% sensitivity and 100% specificity for the assessment of PBH severity (*No PBH*: LBGI_FGM_GT ≤ 4.6; *PBH*: LBGI_FGM_GT > 4.6). *PBH* post-bariatric hypoglycaemia, *ROC* receiver operating characteristic, *LBGI*_*FGM*_*GT* low blood glucose index (adjusted), *AUC* area under the curve.

In contrast, glucose deviations from target range were significantly different between groups (average daily risk ratio [adjusted] [ADRR_FGM_GT]), with *PBH* sub-group presenting significant deviations from target range that were more pronounced towards the low glucose range (low blood glucose index [adjusted] [LBGI_FGM_GT]) rather than to the high glucose range (High Blood Glucose Index [adjusted] [HBGI_FGM_GT]) (Table 2, Fig. 1a).

Additionally, LBGI_FGM_GT as a surrogate of hypoglycaemia risk proved to be an excellent index to confirm PBH using a cut-off value of over 4.6 (area under the receiver operating characteristic [ROC] curve [AUC] = 1.000, standard deviation = 0.000, 95% CI 1.000, 1.000, *p* = 0.003), being 100% sensitive and 100% specific for PBH in individuals with compatible symptoms (Fig. 1b, Supplementary Table S1 online).

### Detailed case descriptions

Two patients with obesity without type 2 diabetes underwent a standardized RYGB procedure performed by the same surgeons and were followed-up by the same multidisciplinary team. After undergoing RYGB, both subjects achieved a successful weight reduction of over 50% of excess weight (excess Body Mass Index [BMI] loss [%EBMIL]: A: 99.4%; B: 52.9%) with improvement of comorbid conditions. Both subjects spontaneously reported the onset of symptoms suggestive of hypoglycaemia over 4 years after surgery, with no evidence of glucose intolerance or type 2 diabetes while off any drugs with glucose-lowering potential (glycated haemoglobin [HbA_1c_]: A: 4.9%; B: 5.3%) (Table 3).

**Table 3:** Patients’ demographics, anthropometrics and biochemical profile.

	Patient A	Patient B
**Demographic features**		
Age (years)	37	52
Sex (F/M)	F	F
**Anthropometric features**		
Time since RYGB (years)	4.8	4.0
Symptom onset (years after RYGB)	~ 4–5	3.4
Pre-operative weight (kg)	86	108
Post-operative weight (kg)	61	84
Pre-operative BMI (kg/m^2^)	35.3	43.3
Post-operative BMI (kg/m^2^)	25.1	33.6
EBMIL (%)	99.4	52.9
**Biochemical profile**		
HbA_1c_ (%/mmol/mol)	4.9/30	5.3/34
Glucose (mg/dl/mmol/l)	87/4.8	92/5.1
Insulin (pmol/l)	41.0	71.5
C-peptide (nmol/l)	0.436	0.439
HOMA2-%β (%)	83.9	109.7
HOMA2-%S (%)	132.7	75.1
HOMA2-IR	0.75	1.33

Biochemical measurements were performed in plasma samples obtained after an overnight fast.

*RYGB* Roux-en-Y gastric bypass, *EBMIL* excess BMI loss, *HbA*_*1c*_ glycated haemoglobin, *HOMA2-%β* updated homeostasis model assessment for β-cell function, *HOMA2-%S* updated homeostasis model assessment for insulin sensitivity, *HOMA2-IR* updated homeostasis model assessment for insulin resistance.

Patient A is a 37 years-old Caucasian female that underwent laparoscopic RYGB for the treatment of obesity grade II (BMI 35.3 kg/m^2^) with gastro-oesophageal reflux disease and osteoarthritis (OA) as obesity comorbidities. Almost 5 years after RYGB surgery with a BMI of 25.1 kg/m^2^, unremarkable biochemical and metabolic profiles and fully resolved obesity comorbidities, the patient spontaneously reported during a routine medical appointment the onset of episodic postprandial sweating and near fainting since the past 3 months. At that time, the patient was only under multivitamin supplements (Table 3).

Patient B is a 52 years-old Caucasian female submitted to laparoscopic RYGB for the treatment of obesity grade III (weight 108 kg, BMI 43.3 kg/m^2^), dyslipidaemia and OA, who despite still being obese experienced a 10-point reduction in BMI with normalization of the lipid profile after the procedure. Four years after surgery, the patient was attended at the emergency department for loss of consciousness with hypoglycaemia documented by paramedics. Past medical history was otherwise irrelevant, apart from primary hypothyroidism diagnosed 20-years earlier for which she was under levothyroxine replacement therapy with a normal thyroid function in addition to the multivitamin supplements (Table 3).

Both patients depicted surrogate measures (updated homeostasis model assessment indexes [HOMA2]) of preserved beta cell function (HOMA2-%β: A: 83.9%; B: 109.7%; reference value: > 72.5% for female^18^) and normal peripheral insulin resistance (HOMA2-IR: A: 0.75; B: 1.33; reference value: < 1.41 for female^18^) and sensitivity (HOMA2-%S: A: 132.7%; B: 75.1%; reference value: > 63.7% for female^18^) (Table 3).

Analysing the FSD and FGM records for similar time periods (patient A: 11.87 days; patient B: 11.88, Table 4), patient A and patient B had 3 and 12 low glucose events (LGEs), respectively. In both patients, LGE occurred in the postprandial period with a 60 to 90 min interval after a glucose excursion (LGE preceded by interstitial fluid glucose [IFG] >140 mg/dl: A: 100% [3 of 3]; B: 83.3% [10 of 12]) leading to either low (LGE with IFG nadir < 54 mg/dl: A: 100% [3 of 3]; B: 91.7% [11 of 12]) or very low (LGE with IFG nadir < 40 mg/dl: A: 0% [0 of 3]; B: 33.3% [4 of 12]) glucose nadirs (Table 4). Paired FSD and FGM data analysis revealed that patient A and B experienced 1 of 3 and 3 of 12 symptomatic LGE, respectively. Symptomatic reports not matching LGE were exclusively reported by patient B (2 of 5). Symptoms were either neuroglycopenic (A: none; B: 2) or neuroglycopenic plus autonomic (A: 1; B: 3), with none of the patients reporting the exclusive occurrence of autonomic symptoms (Table 4).

**Table 4:** Flash glucose monitoring (FGM) data analysis and symptom events reported.

	Patient A	Patient B
**Events report**		
LGE (n)	3	12
Preceded by IFG > 140 mg/dl	3	10
IFG nadir < 54 mg/dl	3	11
IFG nadir ≤ 40 mg/dl	0	4
IFG maximal excursion (mg/dl)	111.7 ± 2.4	130.1 ± 18.7
Time from IFG peak to nadir (minutes)	69.7 ± 4.4	90.9 ± 6.0
Symptoms concurrent with LGE (n)	1 of 1	3 of 5
Only autonomic	0 of 0	0 of 0
Only neuroglycopenic	0 of 0	1 of 2
Autonomic and neuroglycopenic	1 of 1	2 of 3
**FGM evaluation analysis**		
Duration (days)	11.87	11.88
Valid readings (N)	1,099	1,129
Data capture rate (%)	97.44	99.55
IFG (mg/dl)	90 (79–111)	88 (78–114)
Time IFG > 140 mg/dl (%)	7.23	11.99
Time IFG 70–140 mg/dl (%)	89.81	77.48
Time IFG < 70 mg/dl (%)	2.95	10.53
Time IFG < 54 mg/dl (%)	0.00	2.92
MAG change (mmol/l × h^−1^)	2.7	3.0
LBGI_FGM_GT	2.3	5.2
HBGI_FGM_GT	2.4	3.3
ADRR_FGM_GT	50.2	67.1
CONGA1	2.4	2.9
MODD	1.2	1.6

Results are presented as median (interquartile range) and mean ± standard error of the mean.

*LGE* low glucose event, *IFG* interstitial fluid glucose, *MAG* *change* mean absolute glucose change, *LBGI*_*FGM*_*GT* low blood glucose index (adjusted), *HBGI*_*FGM*_*GT* high blood glucose index (adjusted), *ADRR*_*FGM*_*GT* average daily risk ratio (adjusted), *CONGA1* continuous overlapping net glycaemic action, *MODD* mean of daily differences.

**Figure 2:**
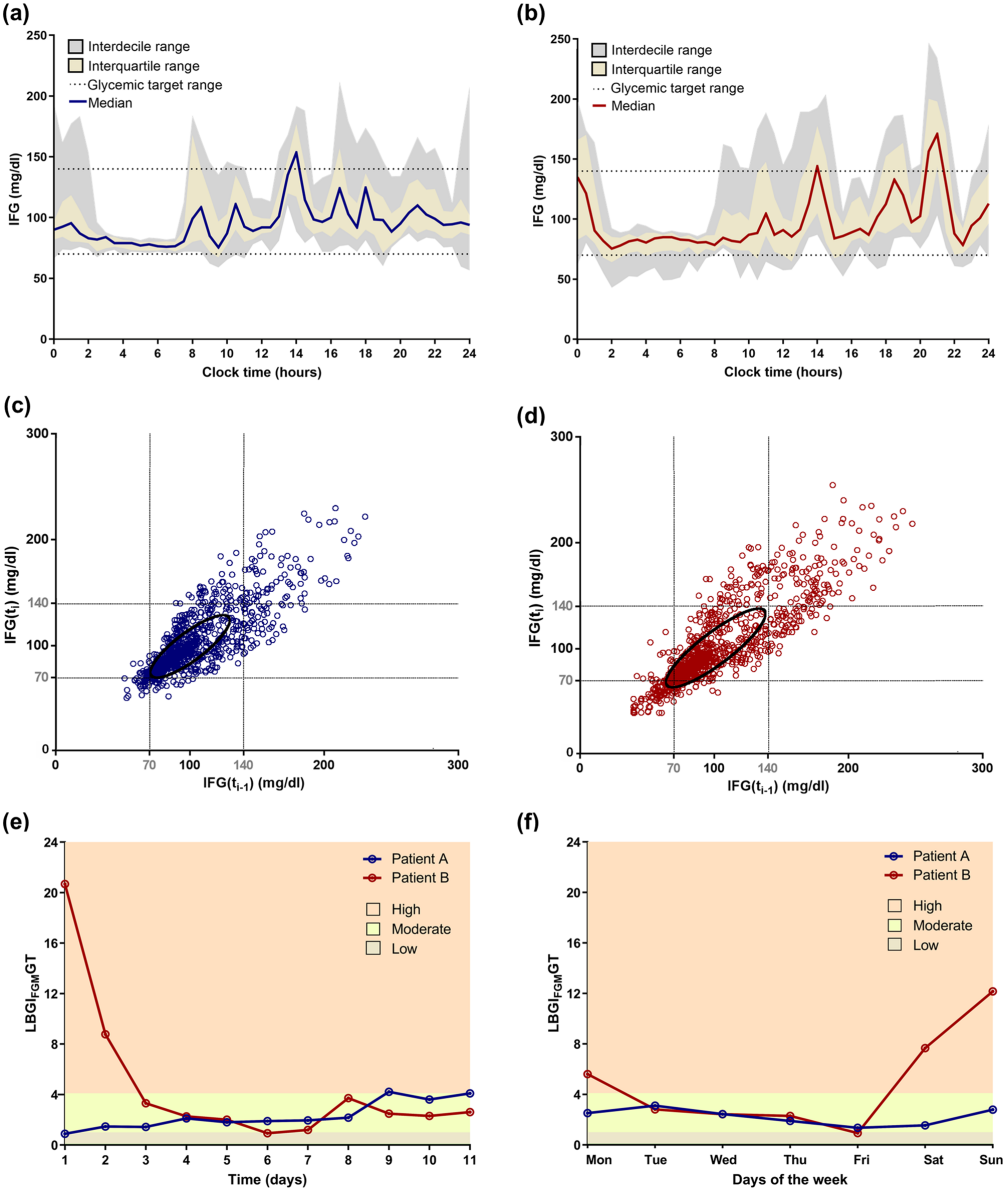
Flash glucose monitoring data graphical illustration. Daily glucose profile (**a**,**b**) and Poincaré plot (**c**,**d**) of patients A (**a**,**c**) and B (**b**,**d**) and hypoglycaemia risk per day (**e**) and per weekday (**f**). Poincaré plots (**c**,**d**) relate each record (IFGt_i_) with the previous one (IFGt_i−1_). Glycaemic target range (70–140 mg/dl) is marked in dots (**a**–**d**) and hypoglycaemia risks are illustrated by different colours (**e**,**f**) low 0.1–1.0; moderate 1.0–4.1; high > 4.1. *IFG* interstitial fluid glucose, *LBGI*_*FGM*_*GT* Low Blood Glucose Index (adjusted).

Greater glucose excursions were observed in patient B (IFG maximal excursion: A: 111.7 ± 2.4 mg/dl; B: 130.1 ± 18.7 mg/dl) and lead to longer lasting glucose fluctuations (time from IFG peak to nadir: A: 69.7 ± 4.4 min; B: 90.9±6.0 min) (Table 4). Central tendency measures of IFG records, median and interquartile range (A: 90 [79–111] mg/dl; B: 88 [78–114] mg/dl), depicted no apparent differences between study subjects. Both patients presented a higher glucose variability during daytime as compared to overnight periods (Fig. 2a,b) along with overall similar intra-daily glucose variability (CONGA1: A: 2.4; B: 2.9) (Table 4).

The deviations from target glucose range (%time out of range: A: 10.19%; B: 22.52%; ADRR_FGM_GT: A: 50.2; B: 67.1), including percentage of time above (A: 7.23%; B: 11.99%) or below target (Time IFG < 70 mg/dl: A: 2.95%; B: 10.53%; Time IFG < 54 mg/dl: A: 0.00%; B: 2.92%), and risks of high (HBGI_FGM_GT: A: 2.4; B: 3.3) or low glucose (LBGI_FGM_GT: A: 2.3; B: 5.2) diverged between the two patients (Table 4). A higher dispersion of IFG consecutive records, suggestive of fast short-term glucose fluctuations (MAG change: [A: 2.7; B: 3.0; reference values: 0.5–2.2] mmol/l × h^−1^^17^), was observed in both post-RYGB patients, although higher for Patient B (Fig. 2c,d) (Table 4).

This trend is illustrated by the Poincaré plots with greater dispersion of values towards both low and high glucose levels and around the x = y line, depicting the higher tendency for fast glucose changes and risk of hypoglycaemia in Patient B, despite the similitude of central tendency measures represented by the ellipses (mean ± SD: A: 100 ± 30 mg/dl; B: 100±38) (Fig. 2c,d).

Summary measures of inter-daily glucose variability (MODD: A: 1.2; B: 1.6) were not visibly different between the two patients (Table 4). However, graphical representation of hypoglycaemia risk throughout the monitoring period depicted a moderate hypoglycaemia risk for Patient A and a higher hypoglycaemia risk in the initial days of FGM for Patient B (Fig. 2e,f). These findings were similar to those observed in study subgroup analysis (Table 2).

## Discussion

Herein we describe 13 patient cases presenting with PBH^5–8^ and two of them in further detail. PBH is a condition for which there are no established diagnosis criteria nor recommended investigation protocols^8^. The only consensus is that hypoglycaemia as cause of patient symptoms must be confirmed in accordance to the Whipple triad^8^ and other endocrine and non-endocrine causes for hypoglycaemia, including insulinoma and glucose lowering drugs, should be ruled out in patients with past-medical history of bariatric surgery before assuming the diagnosis of PBH. Since patients typically present with normal fasting glucose and postprandial hypoglycaemia that occurs most often 60 to 180 min after a meal^8,14^, the use of provocative tests with oral glucose^7^ or liquid^13^ and even solid^19^ mixed meals as tools to diagnose PBH has been proposed. However, these provocative tests represent an artificial scenario and a positive test with reactive hypoglycaemia is often observed in post-bariatric patients without further evidence of the condition, thus overestimating PBH diagnosis^8^. Therefore, given the aforementioned unmet needs, we hypothesized that a FSD coupled with FGM could be a useful tool for diagnosis and risk stratification of patients presenting clinical features suggestive of PBH.

To test this hypothesis, patients were requested to record food intake and symptoms experienced while monitoring IFG with the commercially available FGM system instead of using a continuous glucose monitoring (CGM) system. The medical device used in this study allows to retrieve CGM-like data with an overall good accuracy, including over periods of fast glycaemic excursions, despite being slightly less accurate in the low glucose range and having a relatively high lower limit of detection (2.2 mmol/l, 40 mg/dl) when compared to other glucose monitoring systems^20,21^, therefore carrying the potential risk of underestimating the occurrence of severe hypoglycaemia. For the purpose of this study, using the FGM system had the advantages of being widely available in community pharmacies, relatively inexpensive, easy to use, not requiring finger prick calibration and having a patient-friendly software^20^. Furthermore, as the FGM device used does not provide hypo or hyperglycaemic alerts, thus avoiding a source of bias raised by hypoglycaemia awareness generated by system alarms instead of driven by patient symptoms^20^.

Paired analysis of symptom entries and FGM data made evident the discrepancy between symptoms and IFG levels, with symptoms compatible with hypoglycaemia not always matching low IFG. This came as no surprise, since hypoglycaemia symptoms are highly unspecific and overlap with those typical of dumping syndrome, a common complication of upper gastrointestinal surgery^14^. This finding highlights the potential of this protocol for screening patients with suspected PBH in order to document hypoglycaemia as the cause of patient symptoms, while avoiding further investigation if not confirmed.

A more detailed analysis of FSD and IFG records allowed to disclose the key clinical elements of PBH, namely the occurrence of postprandial LGE after a high glucose excursion, along with reassuring steady overnight and fasting glucose profiles. This glucose pattern tends to characterize PBH, in contrast to other conditions presenting with fasting hypoglycaemia, such as insulinomas^8,11,13,14^.

Targeted FGM data analysis provided further insights into glucose dynamics. Summary measures, such as mean and standard deviation, but also median, percentile distribution and the time spent in each glycaemic range are widely available since these are computed by FGM and CGM devices’ software. However, these summary measures often fail to depict glycaemic fluctuation trends^22^. In contrast, our data analysis protocol provides additional insights into glycaemic variability patterns, including speed of glycaemic variation (MAG change), overall magnitude of deviations towards hyper and hypoglycaemic range (HBGI_FGM_GT and LBGI_FGM_GT respectively) and daily patterns (ADRR_FGM_GT and MODD). These parameters revealed to be significantly different in our study sub-group subjects. Moreover, assessing glycaemic variability is clinically relevant since it can be associated with adverse cardiovascular outcomes^23^.

In fact, MAG change allowed to document the presence of high glucose variability^17^. Indeed, high glycaemic variability has been broadly reported after RYGB^24,25^ in addition to rapid glycaemic fluctuations^26^ towards both hyper and hypoglycaemia (ADRR_FGM_GT^27^), which are well recognized risk factors for PBH^13,28^. Moreover, the percentages of time out of glucose target range and the risk variables computed with FGM retrieved data (LBGI_FGM_GT^29,30^, HBGI_FGM_GT^29,30^ and ADRR_FGM_GT^27^) revealed two distinct hypoglycaemia risk profiles. A higher LBGI_FGM_GT^29,30^ or HBGI_FGM_GT^29,30^ reflect a greater tendency for hypoglycaemia or hyperglycaemia even if not consummated, which is clinically relevant when considering primary prevention. Furthermore, intra-daily overall glucose variability^26^ (CONGA1^31^) matched the glucose unpredictability^32^, as graphically suggested by the daily glucose profiles curves and Poincaré plots, with greater glucose excursions during daytime as compared to the more steady glucose values observed overnight as previously reported^13^. This occurred despite the apparent absence of relevant inter-daily glycaemic profiles variability (MODD^33^).

In addition, FGM data also enabled to assess the frequency and severity of LGE. In particular, this allowed to differentiate the profiles of patient A and B, with patient B experiencing more frequent and severe hypoglycaemic events. Noteworthy, the same patient had a higher BMI and a lower %EBMIL. Despite some evidence suggesting that post-RYGB patients with lower BMI are more likely to experience hypoglycaemia^16,34^, overeating to correct or prevent hypoglycaemia with subsequent weight gain is a well-known phenomenon among patients with diabetes, described in PBH as well^6,35^. A mismatch between symptoms and frequency or severity of hypoglycaemia was also observed, as the patient with the most severe and recurrent LGE was proportionally the least symptomatic, thus suggesting that frequent hypoglycaemic events also raise the risk of hypoglycaemia unawareness in patients without diabetes^8,15^.

Moreover, from glucose profile analysis, it was also noticeable that the risk of hypoglycaemia in the patient with the more severe PBH profile was higher in the first days of FGM monitoring. Long-term usage of FGM systems was reported to decrease the occurrence of hypoglycaemia that has been attributed to increased self-awareness over glucose fluctuations, ultimately leading to patient behaviour modification^36^. Thus, our data further supports the potential use of this protocol for PBH management by promoting patient elicited behavioural changes and modification of eating habits and eventually by allowing tailored dietary interventions by healthcare practitioners.

Noteworthy is the fact that the aforementioned indexes of glycaemic variability that allowed the detailed characterization of the patients’ glucose profile are the end result of the calculation of new mathematical models after the originally described in the literature^30^ for symmetrizing glucose values, which resulted in new risk variables (LBGI_FGM_GT, HBGI_FGM_GT and ADRR_FGM_GT). The mathematical function was recalculated to match the glucose limits of the device (40–500 mg/dl; 2.2–27.8 mmol/l)^20^, with a higher lower detection limit of 40 mg/dl (2.2 mmol/l) than the 20 mg/dl (1.1 mmol/l) considered in the original model and a lower upper detection limit of 500 mg/dl (27.8 mmol/l), against the original one (600 mg/dl; 33.3 mmol/l). This model adjustment was necessary to prevent underestimation of hypoglycaemia and hyperglycaemia risks. Additionally, the target glucose range was adjusted to the physiological glucose range (70–140 mg/dl; 3.9–7.8 mmol/l), since this differs from the target glucose range used in the original model for patients with diabetes (70–180 mg/dl; 3.9–10.0 mmol/l)^30^.

To the best of our knowledge, this proof-of-concept study is the first to demonstrate the potential use of the FGM technology for the differential diagnosis and management of PBH. Besides enabling PBH diagnosis, this protocol has potential for fine tuning patient care by allowing risk stratification and setting the grounds for improved clinical management. Nevertheless, this protocol still requires further validation by assessing glycaemic dynamics in different bariatric patient populations, including matched asymptomatic surgical and non-surgical controls in order to validate the metrics herein computed in addition to its added value as a diagnostic tool.

PBH remains an unmet clinical challenge. The use of a FSD coupled with FGM for clinical assessment of patients with suspected PBH including the usefulness of LBGI_FGM_GT index as a diagnostic tool was herein demonstrated. Our results highlight that a user-friendly and easy to implement study protocol followed by targeted data analysis is able to retrieve relevant insights towards PBH diagnosis, grading or even patients’ management, by eliciting modifications of patient habits and allowing tailored dietary intervention.

## Methods

### Subject selection and study protocol

Subjects (N=13) previously submitted to RYGB surgery that self-reported symptoms suggestive of hypoglycaemia were enrolled on convenience basis, as first come first offer, to undergo a comprehensive clinical assessment to confirm or exclude the PBH, in line with our previous studies^37^. Two patient cases are reported in further detail to illustrate the clinical utility of the proposed protocol.

After an initial standard of practice evaluation to exclude other causes of hypoglycaemia, including endocrine disorders and drugs, patients were requested to fill a FSD while using a commercially available FGM system (FreeStyle Libre, Abbott Diabetes Care, Maidenhead, UK)^20^. This FGM medical device performs automatic measurements of IFG every 15 min for 14 consecutive days corresponding to the lifespan of a single disposable sensor. In addition, the device can provide estimates of IFG whenever the patient forces a reading^20^.

Subjects were given instruction on how to fill the FSD by providing information on time of onset and symptoms description in as much detail as possible and to scan the FGM sensor every 6 h to minimize data loss, as the maximum sensor storage capacity is 8 h. Entries of palpitations, tremor, anxiety, sweating, hunger or tingling/paraesthesia were classified as autonomic symptoms, whereas visual disturbances, headaches, weakness, slurred speech, confusion, concentration difficulties, drowsiness, altered consciousness or seizures were classified as neuroglycopenic symptoms, in line with previous position statements^8^.

This study protocol was submitted and approved by the Hospital Ethics Committee (*Comissão de Ética do Centro Hospitalar Entre o Douro e Vouga*) in compliance with the ethical standards of the *World Medical Association Declaration of Helsinki—Ethical Principles for Medical Research Involving Human Subjects*. Informed written consent was obtained from subjects before undertaking any study procedure. Additional consent to publish identifying information was also obtained from each individual participant.

### Study sub-groups

Study subjects were divided into two sub-groups of patients according to FGM profile in *No PBH* (IFG < 54 mg/dl < 1%) and *PBH* (IFG < 54 mg/dl ≥ 1%), based on established criteria for level 2 hypoglycaemia^38^.

### Biochemical measurements

Updated homeostasis model assessment indexes (HOMA2) values were calculated using fasting glucose and insulin levels assessed in the same day or within the previous 3 months of FGM fitting. Venous blood was collected from the antebrachial vein after a minimum 8 h overnight fast into EDTA tubes (S-Monovette 9.0 ml, K2 EDTA Gel, 1.6 mg/ml, Sarstedt). Blood glucose was measured in whole blood with a glucometer and converted into plasma glucose using the WHO conversion factor of 1.12 (FPG = WBG × 1.12)^39^. Plasma insulin levels were measured by electrochemiluminescence sandwich immunoassay (ECLIA) (Cobas 8000, model e602, Roche Diagnostics, USA), against liquid human serum-based controls: Liquichek Immunoassay Plus Control, Level 1 #361 and Level 3 #363, Bio-Rad.

### Data analysis and mathematical modelling

Subjects’ data was retrieved from our clinical register and included age, gender, type of bariatric surgery procedure performed, comorbidities before the surgery, anthropometrics, biochemical profile routinely performed before and after surgery as standard of care and time elapsed since surgery until symptoms onset.

Percentage of excess BMI loss (EBMIL) was determined as EBMIL (%) = (preoperative BMI – BMI at FGM evaluation)/(preoperative BMI – 25) × 100. HOMA2 were calculated using the HOMA Calculator version 2.2.3 (https://www.dtu.ox.ac.uk, accessed April 2019) as surrogate measures of beta cell function (HOMA2-%β) and peripheral insulin sensitivity (HOMA2-%S) and resistance (HOMA2-IR).

Upon completion of the monitoring period, the sensor was removed and FGM raw data downloaded for targeted analysis. To ensure maximal data accuracy, including study participants’ adaptation to the device and equally timed records, IFG measurements during the first 48 h of monitoring and estimated glucose values provided by forced readings on patient demand were excluded from statistical analysis.

The glucose range target was set between 70 and 140 mg/dl (3.9–7.8 mmol/l) and the percentage of time under each glycaemic range (< 54; < 70; [70–140]; > 140 mg/dl; < 3.0; < 3.9; [3.9–7.8]; > 7.8 mmol/l) was determined by adding all the periods of at least two consecutive reads (duration ≥15 min) in each interval, therefore excluding all isolated reads corresponding to brief deviations lasting less than 15-min long towards another interval. Time gaps derived from IFG data loss due to overridden memory capacity, which is limited to 8 h without the need for sensor scanning, were taken into account and were not included to calculate the percentage of time spent in each glucose range.

A LGE was considered whenever a IFG < 70 mg/dl (< 3.9 mmol/l; hypoglycaemia alert or level 1 hypoglycaemia) with concurrent hypoglycaemia symptoms within a time lag between symptoms and IFG < 70 mg/dl of ± 30 min or whenever a IFG < 54 mg/dl (< 3.0 mmol/l; clinically substantial hypoglycaemia or level 2 hypoglycaemia) was recorded independently of symptoms, in accordance with the thresholds recommended by the American Diabetes Association^38^ and with the international consensus on CGM data interpretation^40^. LGEs were manually recorded to reduce the risk of bias, since patients with PBH often experience consecutive LGEs triggered by successive meals as attempts to correct a first hypoglycaemic episode that eventually result in “rebound hypoglycaemia”. In an automated data analysis process, these would be considered a single protracted LGE, while manual data analysis allows to disclose consecutive LGEs.

Median and interquartile range of the IFG data retrieved by FGM were determined as traditional central tendency measures suitable to describe non-Gaussian distributed data as continuous glucose patterns (Table 5)^22^. For mathematical computation of the FGM data, MAG change^17,41^, CONGA1^31^ and MODD^33^ were calculated as previously described, to outline short-term, hourly and inter-daily glycaemic variability respectively^26^ (Table 5).

**Table 5:** Metrics applied for flash glucose monitoring (FGM) retrieved data analysis.

Metric	Formula applied	Variables	Interpretation
MAG change^17, 41^	$MAG \;change = \frac{{\mathop \sum \nolimits_{t = 1}^{t} \left| {G_{t} - G_{t - 1} } \right|}}{\Delta t}$	$G_{t}$*—*IFG record at time t, in mmol/l $\Delta t$—Time interval, in hours	Timing of IFG fluctuations, with target on short-term glucose variability
CONGA1^31^	$CONGA1 = \sqrt {\frac{{\mathop \sum \nolimits_{t = 1}^{{t_{k} }} \left( {D_{t} - \overline{D}} \right)^{2} }}{k - 1}} ,$ $t > 60$ with $\overline{D} = \frac{{\mathop \sum \nolimits_{t = 1}^{{t_{k} }} D_{t} }}{k}$ and $D_{t} = G_{t} - G_{t - 60}$	$k$*—*Number of IFG records where there is an IFG record 1 h (60-min) before $G_{t}$*—*IFG record at time t, in mmol/l	Hourly glucose variability. As IFG records are 15 min apart, CONGA1 accounts overlapping 60-min periods
MODD^33^	$MODD = \frac{1}{s}\mathop \sum \limits_{{t = t_{1} }}^{{t_{s} }} \left| {G_{t} - G_{t - 1440} } \right|,$ $t > 1,440$	$s$*—*Number of IFG records where there is an IFG record 24-h (1,440 min) before $G_{t}$*—*IFG record at time t, in mmol/l	Inter-daily glucose variation, disclosing circadian glucose trends and periodic patterns
LBGI_FGM_GT and HBGI_FGM_GT^29, 30^	$LBGI_{FGM} GT = \frac{1}{N}\mathop \sum \limits_{i = 1}^{n} rl\left( {G_{i} } \right)$ $HBGI_{FGM} GT = \frac{1}{N}\mathop \sum \limits_{i = 1}^{n} rh\left( {G_{i} } \right)$ $$rl\left( {G_{t} } \right) = \left\{ {\begin{array}{*{20}l} {10 \times f\left( {G_{t} } \right)^{2} } \hfill & {if f\left( {G_{t} } \right) < 0} \hfill \\ 0 \hfill & {otherwise} \hfill \\ \end{array} } \right.$$ $$rh\left( {G_{t} } \right) = \left\{ {\begin{array}{*{20}l} {10 \times f\left( {G_{t} } \right)^{2} } \hfill & {if f\left( {G_{t} } \right) > 0} \hfill \\ 0 \hfill & {otherwise} \hfill \\ \end{array} } \right.$$ $f\left( {G_{t} } \right) = 30.3082 \times \left\{ {\left[ {ln\left( {G_{t} } \right)} \right]^{0.1356} - 1.0726} \right\}$	$N$*—*Total number of IFG records $G_{t}$*—*IFG record at time t, in mmol/l	Risk indexes for predicting deviations towards low (LBGI_FGM_GT) and high (HBGI_FGM_GT) glucose values (adjusted*)
ADRR_FGM_GT^27^	$ADRR_{FGM} GT = \frac{1}{M}\mathop \sum \limits_{i = 1}^{m} \left[ {LR^{i} + HR^{i} } \right]$ with $LR^{i} = \max \left[ {rl\left( {G_{1}^{i} } \right), \ldots ,rl\left( {G_{t}^{i} } \right)} \right]$ and $HR^{i} = \max \left[ {rh\left( {G_{1}^{i} } \right), \ldots ,rh\left( {G_{t}^{i} } \right)} \right]$ for day $i$; $i = 1,2, \ldots M.$	$M$*—*Number of days of IFG monitorization $G_{t}$*—*IFG record at time t, in mmol/l	Extreme deviations from target glucose range towards both high and low glucose levels in each day of IFG evaluation (adjusted*)

*IFG* interstitial fluid glucose, *MAG* *change* mean absolute glucose change, *CONGA1* continuous overlapping net glycaemic action, *MODD* mean of daily differences, *LBGI*_*FGM*_*GT* Low Blood Glucose Index (adjusted), *HBGI*_*FGM*_*GT* High Blood Glucose Index (adjusted), *ADRR*_*FGM*_*GT* average daily risk ratio (adjusted).

*Classical formulae were “adjusted” to establish risk in glucose-tolerant individuals (target range: 70–140 mg/dl; 3.9–7.8 mmol/l) monitored with FreeStyle Libre, Abbott Diabetes Care, Maidenhead, UK (device range: 40–500 mg/dl; 2.2–27.8 mmol/l).

Additionally, glucose values were computed into low blood glucose index (LBGI)^29,30^, high blood glucose index (HBGI)^29,30^ and average daily risk ratio (ADRR)^27^, aimed to symmetrize glucose records while highlighting glucose deviations from the target range towards hypo-, hyperglycaemia or in both directions, respectively (Table 5), since glycaemic excursions towards hyperglycaemia are much more impactful in central tendency measures than deviations into the hypoglycaemic range. For this, the original risk analysis function used to compute these indexes was adjusted to the specific characteristics of the FGM system used. This classic function (Eq. (1)) was originally modelled using data from patients with diabetes assessed with glucose meters with detection ranges distinct from the currently used FGM system^30,42^. The function was recalculated following the original rationale^42^ to comply with the range of the device used (40–500 mg/dl; 2.2–27.8 mmol/l)^20^ and physiological glucose range (70–140 mg/dl; 3.9–7.8 mmol/l), which resulted in Eq. (2) (rational detailed in Table 6) and in adjusted indexes (LBGI_FGM_GT, HBGI_FGM_GT and ADRR_FGM_GT) (Table 5).

1 $${\text{f}}\left( {\text{x}} \right) = 1.794 \times \left\{ {\left[ {{\ln}\left( {\text{x}} \right)} \right]^{1.026} - 1.861} \right\}$$

2 $${\text{f}}\left( {\text{x}} \right) = 30.3082 \times \left\{ {\left[ {{\ln}\left( {\text{x}} \right)} \right]^{0.1356} - 1.0726} \right\}$$

**Table 6:** Calculation of the risk analysis function analogous to originally described^30^.

Rational	Details
Premises	**Target glucose range for glucose-tolerant individuals** 3.9–7.8 mmol/l (70–140 mg/dl) **Device glucose range (FreeStyle Libre)** 2.2–27.8 mmol/l (40–500 mg/dl)
Clinical assumptions	The transformation should make the device glucose range symmetrical around zero The transformation should make the target glucose range symmetrical around zero The transformed values should have as range $\left[ { - \sqrt {10} ,\sqrt {10} } \right]$, yielding a final risk that theoretically ranges from 0 to 100
Equations	$f\left( {G_{t} ,\alpha ,\beta ,\gamma } \right) = \gamma \times \left\{ {\left[ {ln\left( {G_{t} } \right)} \right]^{\alpha } - \beta } \right\}, \alpha ,\beta > 0 \quad \left( {{\text{G}}_{{\text{t}}} \;{\text{in mmol}}/{\text{l}}} \right)$ $\left[ {ln\left( {27.8} \right)} \right]^{\alpha } - \beta = - \left\{ {\left[ {ln\left( {2.2} \right)} \right]^{\alpha } - \beta } \right\}$ $\left[ {ln\left( {7.8} \right)} \right]^{\alpha } - \beta = - \left\{ {\left[ {ln\left( {3.9} \right)} \right]^{\alpha } - \beta } \right\}$ $\gamma \left\{ {\left[ {ln\left( {27.8} \right)} \right]^{\alpha } - \beta } \right\} = - \gamma \left\{ {\left[ {ln\left( {2.2} \right)} \right]^{\alpha } - \beta } \right\} = \sqrt {10}$
Results	${\text{If}}\;\alpha > 0,$ $$\left\{ {\begin{array}{*{20}c} { \alpha = 0.1356} \\ {\beta = 1.0726} \\ { \gamma = 30.3082} \\ \end{array} } \right.$$
Function	${\varvec{f}}\left( {{\varvec{G}}_{{\varvec{t}}} } \right) = 30.3082 \times \left\{ {\left[ {{\varvec{ln}}\left( {{\varvec{G}}_{{\varvec{t}}} } \right)} \right]^{0.1356} - 1.0726} \right\},\;{\mathbf{G}}_{{\mathbf{t}}} {\mathbf{in}} \, {\mathbf{mmol}}/{\mathbf{l}}$ ${\varvec{f}}\left( {{\varvec{G}}_{{\varvec{t}}} } \right) = 30.3082 \times \left\{ {\left[ {{\varvec{ln}}\left( {{\varvec{G}}_{{\varvec{t}}} /18.016} \right)} \right]^{0.1356} - 1.0726} \right\},\;{\mathbf{G}}_{{\mathbf{t}}} {\mathbf{in}} \, {\mathbf{mg}}/{\mathbf{dl}}$

Solution by Newton’s method. Abbreviations: G_t_ interstitial fluid glucose values recorded by FreeStyle Libre, Abbott Diabetes Care, Maidenhead, UK.

### Graphical and statistical analysis

Ellipses in Poincaré plots were computed with centre on mean glucose and standard deviation as axes to depict overall glucose fluctuation^32^. Raw data was analysed using IBM SPSS Statistics Version 25 for Windows, for percentiles; GNU Octave version 4.4.0, for Poincaré plot computation; GraphPad Prism version 8.0.1 for Windows, GraphPad Software, San Diego, California USA, www.graphpad.com, for all other illustrations; and Excel 2016, Microsoft Office 365 ProPlus, for remaining calculations.

Data was independently analysed by two double-blinded researchers (CBL and SSP) and cross-matched for verification and validation of the protocol hereby summarized, with no differences found in the results.

For group data analysis, variables are expressed as median (interquartile range). Variables were assumed to be non-normally distributed due to small sample size and groups were compared using Mann-Whitney test. Categorical variables are represented as proportions and were compared using Fisher’s exact test. The differences between the two sub-groups were considered statistically significant when two-tailed *p* value was below 0.05.

The area under the ROC curve was used to determine the power of LBGI_FGM_GT to estimate PBH. Based on the AUC of the ROC curve, a diagnostic tool can be considered excellent (for values ranging from 0.90 to 1.00), good (0.80 to 0.90), fair (0.70 to 0.80), poor (0.60 to 0.70) or fail (below 0.60)^43^.

## Supplementary information

Supplementary file1

## Data Availability

The datasets generated during and/or analysed during the current study are available from the corresponding author on request.
