# Supplementary material for: Use of flash glucose monitoring for post-bariatric hypoglycaemia diagnosis and management
Source: Sci Rep. 2020 Jul 6;10:11061. doi: 10.1038/s41598-020-68029-8 (PMC7338422; doi:10.1038/s41598-020-68029-8)
Supplement: Supplementary file 1 — Supplementary file1 [file 41598_2020_68029_MOESM1_ESM.pdf]

Use of flash glucose monitoring for post-bariatric hypoglycaemia diagnosis and management

Carolina B. Lobato[1,2], Sofia S. Pereira[1,2], Marta Guimarães[1-3], Tiago Morais[1,2], Pedro Oliveira[4], Jorge P. M. de Carvalho[5], Mário Nora[1,3], and Mariana P. Monteiro[1,2,*]

[1]Endocrine, Cardiovascular & Metabolic Research, Unit for Multidisciplinary Research in Biomedicine (UMIB), University of Porto, Porto, 4050-313, Portugal
[2]Department of Anatomy, Institute of Biomedical Sciences Abel Salazar (ICBAS), University of Porto, Porto, 4050-313, Portugal
[3]Department of General Surgery, Centro Hospitalar de Entre o Douro e Vouga, Santa Maria da Feira, 4520-211, Portugal
[4]Department of Population Studies, ICBAS, University of Porto, Porto, 4050-313, Portugal
[5]Department of Mathematics, Faculty of Sciences, University of Porto, Porto, 4169-007, Portugal
[*]mpmonteiro@icbas.up.pt

To whom correspondence and reprint requests should be addressed:
Mariana P. Monteiro, MD, PhD
Institute of Biomedical Sciences Abel Salazar - University of Porto,
Jorge Viterbo Ferreira 228, Ed.1, 3rd Floor, 4050-313, Porto, Portugal;
Telephone number: (00351)220428103
E-mail address: mpmonteiro@icbas.up.pt

Supplementary Table S1. ROC curve analysis of the value of $LBGI_{FGM}GT$ (N=13)

ROC curve cut-off value	Sensitivity (%)	95% CI	Specificity (%)	95% CI	Likelihood ratio
>2.6	100.0	]67.6, 100.0[	20.0	]1.0, 62.4[	1.250
>2.9	100.0	]67.6, 100.0[	40.0	]7.1, 76.9[	1.667
>3.1	100.0	]67.6, 100.0[	60.0	]23.1, 92.9[	2.500
>3.6	100.0	]67.6, 100.0[	80.0	]37.6, 100.0[	5.000
>4.6	100.0	]67.6, 100.0[	100.0	]56.6, 100.0[	-
>8.2	85.5	]52.9, 99.4[	100.0	]56.6, 100.0[	-
>11.9	75.0	]40.9, 95.6[	100.0	]56.6, 100.0[	-
>12.6	62.5	]30.6, 86,3[	100.0	]56.6, 100.0[	-
>13.0	50.0	]21.5, 78.5[	100.0	]56.6, 100.0[	-
>14.8	37.5	]13.7, 69.4[	100.0	]56.6, 100.0[	-
>17.5	25.0	]4.4, 59.1[	100.0	]56.6, 100.0[	-
>18.8	12.5	]0.6, 47.1[	100.0	]56.6, 100.0[	-

ROC curve AUC=1.000, SD=0.000, 95% CI=]1.000, 1.000[; p=0.003. At the optimal cut-off value of 4.6, $LBGI_{FGM}GT$ had 100% sensitivity and 100% specificity for the assessment of PBH severity (*No PBH*: $LBGI_{FGM}GT \leq 4.6$; *PBH*: $LBGI_{FGM}GT > 4.6$). Abbreviations: ROC – receiver operating characteristic; CI = Confidence Interval; SD = Standard deviation; PBH – Post-bariatric hypoglycaemia; $LBGI_{FGM}GT$ – Low Blood Glucose Index (adjusted); AUC – Area under the curve.
